# HIV infection and antiretroviral therapy lead to unfolded protein response activation

**DOI:** 10.1186/s12985-015-0298-0

**Published:** 2015-05-15

**Authors:** Mariana Borsa, Pedro L. C. Ferreira, Andrea Petry, Luiz G. E. Ferreira, Maristela M. Camargo, Dumith Chequer Bou-Habib, Aguinaldo R. Pinto

**Affiliations:** Laboratório de Imunologia Aplicada, Departamento de Microbiologia, Imunologia e Parasitologia, Universidade Federal de Santa Catarina, Florianópolis, SC Brazil; Laboratório de Pesquisas sobre o Timo, Instituto Oswaldo Cruz, Rio de Janeiro, RJ Brazil; Centro de Hematologia e Hemoterapia de Santa Catarina, Florianópolis, SC Brazil; Hospital Regional Homero de Miranda Gomes, São José, SC Brazil; Departamento de Imunologia, Instituto de Ciências Biomédicas, Universidade de São Paulo, São Paulo, SP Brazil

**Keywords:** HIV, Aids, UPR pathway, Endoplasmic reticulum stress, Antiretroviral therapy, Cell stress

## Abstract

**Background:**

The unfolded protein response (UPR) is one of the pathways triggered to ensure quality control of the proteins assembled in the endoplasmic reticulum (ER) when cell homeostasis is compromised. This mechanism is primarily composed of three transmembrane proteins serving as stress sensors: PKR-like ER kinase (PERK), activating transcription factor 6 (ATF6), and inositol-requiring enzyme 1 (IRE1). These three proteins’ synergic action elicits translation and transcriptional downstream pathways, leading to less protein production and activating genes that encode important proteins in folding processes, including chaperones. Previous reports showed that viruses have evolved mechanisms to curtail or customize this UPR signaling for their own benefit. However, HIV infection’s effect on the UPR has scarcely been investigated.

**Methods:**

This work investigated UPR modulation by HIV infection by assessing UPR-related protein expression under *in vitro* and *in vivo* conditions via Western blotting. Antiretroviral (ARV) drugs’ influence on this stress response was also considered.

**Results:**

In *in vitro* and *in vivo* analyses, our results confirm that HIV infection activates stress-response components and that ARV therapy contributes to changes in the UPR’s activation profile.

**Conclusions:**

This is the first report showing UPR-related protein expression in HIV target cells derived directly from HIV-infected patients receiving different ARV therapies. Thus, two mechanisms may occur simultaneously: interference by HIV itself and the ARV drugs’ pharmacological effects as UPR activators. New evidence of how HIV modulates the UPR to enhance its own replication and secure infection success is also presented.

**Electronic supplementary material:**

The online version of this article (doi:10.1186/s12985-015-0298-0) contains supplementary material, which is available to authorized users.

## Background

The unfolded protein response (UPR) is a mechanism elicited whenever protein folding is compromised inside the endoplasmic reticulum (ER) [1]. In the last few years, interest in the UPR signaling network has been increasing due to the implication of ER stress mechanisms in a wide range of pathologies, including infections, ischemic injury, neurodegenerative disorders, metabolic diseases, and neoplasias [2, 3]. Under stress conditions, UPR activation is essential to restore protein-folding homeostasis; however, if the damage to the cell is highly severe, the chronic ER stress and UPR signaling lead to cell apoptosis [1]. In mammals in particular, unfolded proteins are recognized in the ER lumen by the chaperone molecule immunoglobulin heavy chain-binding protein (GRP78/BiP), which dissociates from three transmembrane proteins: PKR-like ER kinase (PERK), activating transcription factor 6 (ATF6), and inositol-requiring enzyme 1 (IRE1) [4]. The synergic action of these three proteins, also known as UPR sensors, leads to translation and transcriptional downstream pathways that lead to less protein production and to the activation of genes that encode important proteins in folding processes, such as chaperones [1, 4].

The presence of unfolded proteins inside the ER lumen can result from many different physiological conditions, such as cell development and differentiation, high production of secreted proteins, genetic mutation, or oxidative stress, or may even be a response against certain pathogens, including viral infections [1]. Viral activation of the UPR may be specifically caused by the production of new viral particles due to the high load of viral protein production in the ER during infection. However, certain downstream effectors are not necessarily beneficial for viral replication, e.g., protein degradation by the ubiquitin- and proteasome-dependent process known as ER-associated degradation (ERAD) or apoptosis induction [5, 6]. Nonetheless, viruses are also capable of avoiding activation of several mechanisms in the UPR pathway, which shows their ability to modulate this cellular stress response both to promote cell survival during efficient viral replication and to create an environment more favorable for replication [7].

The effect of HIV infection on the UPR pathway has scarcely been investigated [8, 9]. Regarding HIV target cells in particular, an *in vitro* study using the Jurkat CD4^+^ T-cell line and U1 pro-monocytic cells showed that HIV is responsible for inducing ATF4 transcription and translation, leading to an accumulation of this factor during the acute phase of infection [10]. This report also indicated that ATF4 triggers the reactivation of HIV replication in infected cells through a mechanism mediated by the viral protein Tat, suggesting that the ER stress events that lead to high expression of ATF4 may be relevant for the end of viral latency. Additionally, reports have shown that the protease inhibitors used as part of ARV therapy are responsible for pharmacological activation of the UPR pathway *in vitro* [11–14]. However, despite the known pharmacological effects of ARV drugs against HIV, the possible UPR mechanisms modulated by HIV in the host cell are not completely elucidated.

Thus, we were interested in investigating UPR pathway modulation by HIV infection via assessment of the expression of UPR-related proteins under *in vitro* and *in vivo* conditions. We also considered the influence of ARV drugs on this stress response. This is the first report showing UPR-related protein expression in HIV target cells derived directly from HIV-infected patients receiving different ARV therapies. Here, we specifically report that HIV infection is itself responsible for activating stress-response components and that ARV therapy contributes to a change in the UPR pathway activation profile based on both *in vitro* and *in vivo* analyses.

## Results

### HIV induces BiP expression

**Fig. 1:**
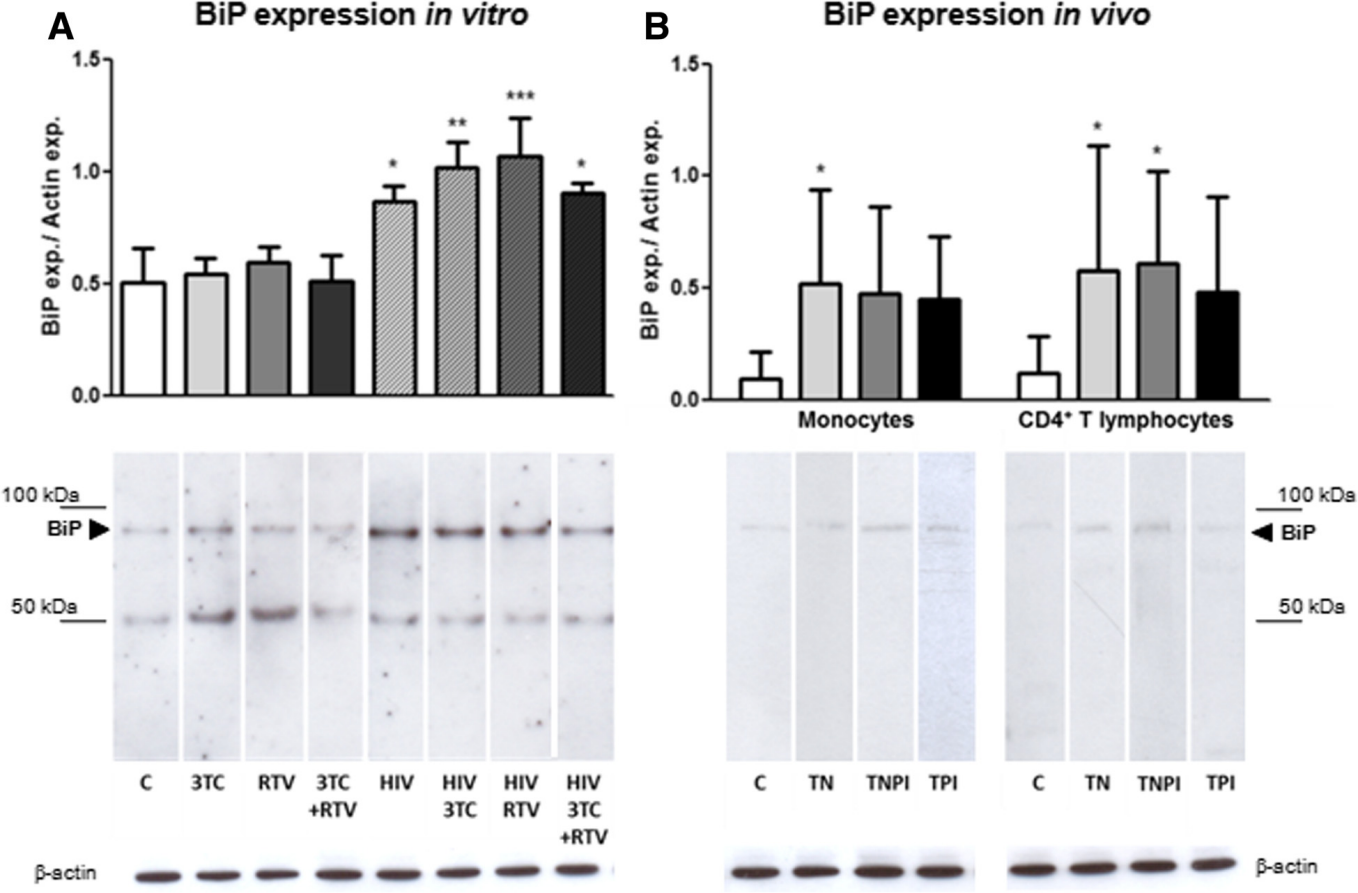
HIV increases BiP expression under both *in vitro* and *in vivo* conditions. The expression of the chaperone BiP (78 kDa) was determined by Western blotting (bottom panels) using an anti-BiP antibody. Actin was used as a loading control. The intensity of the resulting bands was measured using ImageJ, and the BiP/actin ratio is shown in the upper panels. Each lane shown in the Western blots is representative of cells under the same infectious and pharmacological conditions *in vitro* or *in vivo*. The bars represent mean ± standard deviation (SD) of BiP expression within each group. **a** PBMCs were infected with the HIV-1 R5 isolate Ba-L and maintained free of ARV drugs or incubated with a nucleoside reverse transcriptase inhibitor (lamivudine/3TC) and/or a protease inhibitor (ritonavir/RTV) for 7 days, followed by harvesting to prepare protein lysates. C = control (non-infected PBMCs); 3TC = PBMCs incubated with 3TC; RTV = PBMCs incubated with RTV. **b** Enriched monocytes or CD4^+^ T lymphocytes obtained from whole blood from patients/volunteers were used to produce protein lysates. C = control (blood donors), n = 10; TN = the treatment-naïve patients, n = 7; TNPI = patients under ARV therapy without a protease inhibitor, n = 9; TPI = patients under ARV therapy with a protease inhibitor, n = 9. *p < 0.05; **p < 0.01; ***p < 0.001

To determine whether HIV infection induces the chaperone BiP, PBMCs were infected with HIV and then submitted to different ARV treatments (Fig. 1a). The HIV replication in PBMCs from normal donors under different conditions is shown in Additional file 1: Table S1. Furthermore, non-infected cells were submitted to the same ARV treatment conditions to identify any pharmacological modulation of UPR activation. The drugs selected for this study, which were lamivudine (3TC), a nucleoside reverse transcriptase inhibitor, and ritonavir (RTV), a protease inhibitor, were used separately or together. The Western blotting results and further densitometry analysis show that the ARV drugs used did not themselves induce any significant effect on BiP expression in either non-infected or infected clusters. However, infected cells without treatment or under ARV treatment showed significantly higher expression of this chaperone in comparison with non-infected cells, as demonstrated by the densitometry analysis.

The same profile of BiP expression was found when *in vivo* assays were performed using cells from volunteers (Fig. 1b). Both in monocyte- and in CD4^+^ T lymphocyte-enriched populations, the control group, represented by cells from healthy blood donors, showed lower expression of BiP. In contrast, increased expression of this chaperone was observed in HIV-infected cells, and, as already observed in the *in vitro* assays, the type of ARV therapy did not appear to be a determinant of the induction of BiP expression. In monocytes from treatment-naïve HIV patients, BiP expression was significantly higher (*p* < 0.05). CD4^+^ T lymphocytes also presented significantly higher expression of BiP in the treatment-naïve group, similar to expression in cells obtained from HIV-infected patients under treatment without a protease inhibitor (*p* < 0.05). These data suggest that HIV infection is capable of inducing BiP expression under both *in vitro* and *in vivo* conditions, potentially resulting in an environment that favors continued protein folding, which is a significant indication of UPR activation.

### HIV promotes translational attenuation through eIF2α activation

**Fig. 2:**
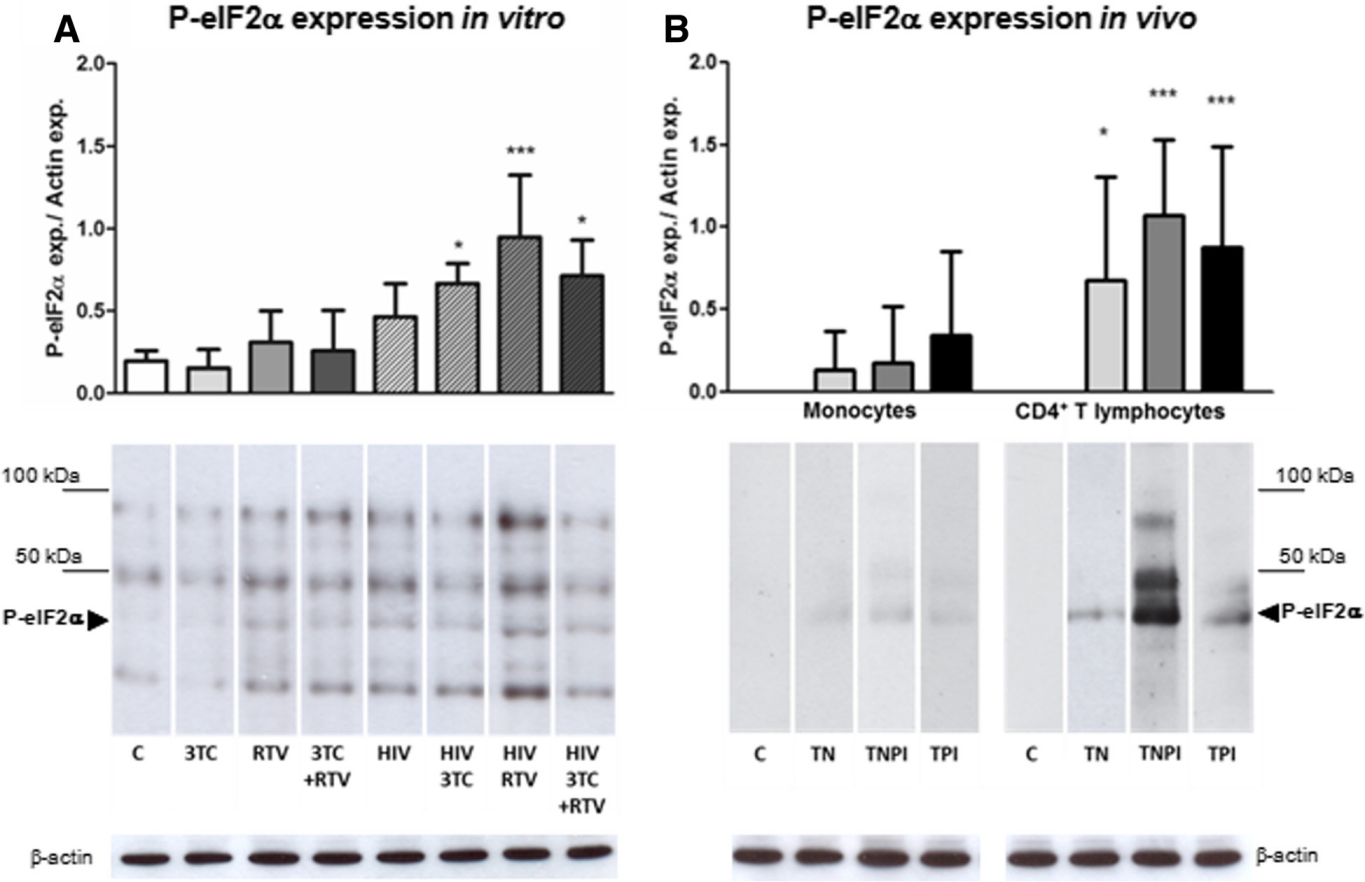
HIV triggers eIF2α phosphorylation. The expression of the transcription factor eIF2α in its phosphorylated form (42 kDa) was determined by Western blotting (bottom panels) using an anti-P-eIF2α antibody. Actin was used as a loading control. The intensity of the resulting bands was measured using ImageJ, and the P-eIF2α/actin ratio is shown in the upper panels. Each lane shown in the Western blots is representative of cells under the same infectious and pharmacological conditions *in vitro* or *in vivo*. The bars represent mean ± standard deviation (SD) of P-eIF2α expression with each group. **a** PBMCs were infected with the HIV-1 R5 isolate Ba-L and maintained free of ARV drugs or incubated with a nucleoside reverse transcriptase inhibitor (lamivudine/3TC) and/or a protease inhibitor (ritonavir/RTV) for 7 days, followed by harvesting to prepare protein lysates. C = control (non-infected PBMCs); 3TC = PBMCs incubated with 3TC; RTV = PBMCs incubated with RTV. **b** Enriched monocytes or CD4^+^ T lymphocytes obtained from whole blood from patients/volunteers were used to produce protein lysates. C = control (blood donors), n = 10; TN = the treatment-naïve patients, n = 7; TNPI = patients under ARV therapy without a protease inhibitor, n = 9; TPI = patients under ARV therapy with a protease inhibitor, n = 9. *p < 0.05; ***p < 0.001

The expression of the phosphorylated form of eIF2α (P-eIF2α) was analyzed under both *in vitro* and *in vivo* conditions by Western blotting. Phosphorylation of eIF2α represents its activation, leading to translational inhibition inside the cell. Under these conditions only, certain specific genes related to the ER stress response do not have their expression attenuated. Furthermore, eIF2α phosphorylation is a consequence of previous PERK activation, suggesting activation of this UPR sensor. In the present study, no difference in P-eIF2α levels was observed in non-infected PBMCs, regardless of the ARV treatment. However, HIV-infected PBMCs that were submitted to ARV treatment showed significantly higher expression of P-eIF2α compared with non-infected cells (Fig. 2a). Interestingly, the highest expression of P-eIF2α was found in cells treated only with ritonavir (*p* < 0.001) compared with the other treatments (*p* < 0.05).

HIV infection was also shown to be an important factor contributing to higher levels of P-eIF2α in cells from HIV patients (Fig. 2b). However, in contrast to what was demonstrated in the *in vitro* assays, the expression of this protein was not detected in non-infected cells from blood donors. Additionally, P-eIF2α expression in the monocyte-enriched subpopulation from HIV-infected volunteers was not significantly higher than in the cells from healthy controls, and the ARV treatment did not seem to be a key factor for this expression. However, in the CD4^+^ T lymphocyte subpopulation, the combination of HIV infection and ARV therapy was shown to be important. In particular, the lymphocytes from HIV-infected patients showed significantly higher expression of P-eIF2α compared with the control group. In this case, as was found in the *in vitro* assays with PBMCs, the ARV therapy led to higher expression of eIF2α (*p* < 0.001) compared with expression in the healthy control group (*p* < 0.05). A comparison between the *in vitro* and *in vivo* results indicated similar dynamics of HIV infection, in which the virus itself seems to be responsible for higher expression of P-eIF2α, but the presence of ARV drugs was also significant. However, the ARV therapy itself was not sufficient to induce the phosphorylation of eIF2α, as indicated by the *in vitro* assays. Therefore, infected PBMCs treated with ARV drugs have a different expression profile than non-infected treated PBMCs do.

### HIV can induce higher levels of IRE1 phosphorylation and activation

**Fig. 3:**
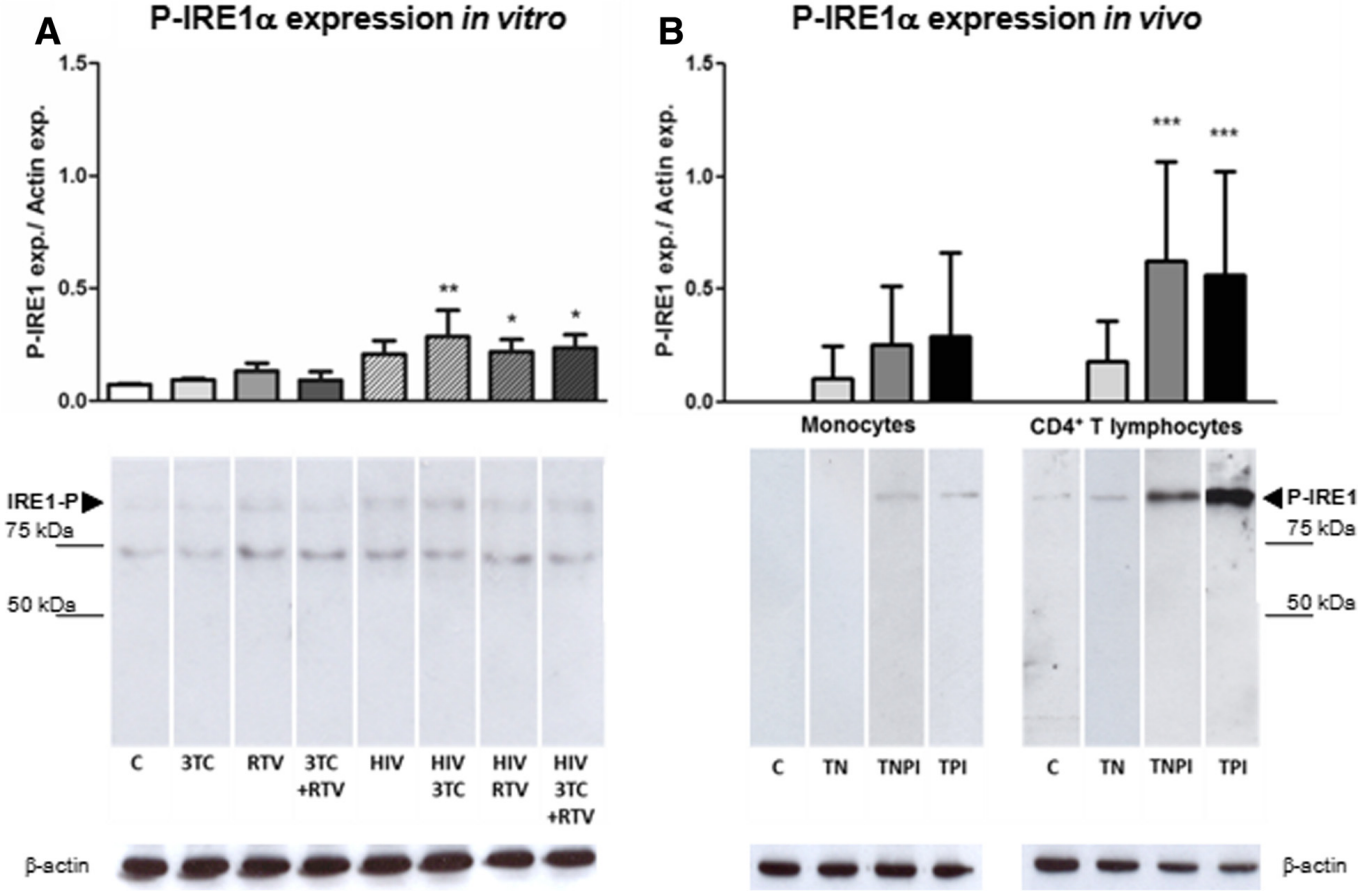
IRE1 shows higher activation in HIV-infected cells. The expression of the UPR sensor IRE1 in its phosphorylated form (100 kDa) was determined by Western blotting (bottom panels) using an anti-P-IRE1 antibody. Actin was used as a loading control. The intensity of the resulting bands was measured using ImageJ, and the P-IRE1/actin ratio is shown in the upper panels. Each lane shown in the Western blots is representative of cells under the same infectious and pharmacological conditions *in vitro* or *in vivo*. The bars represent mean ± standard deviation (SD) of P-IRE1 expression in each group. **a** PBMCs were infected with the HIV-1 R5 isolate Ba-L and maintained free of ARV drugs or incubated with a nucleoside reverse transcriptase inhibitor (lamivudine/3TC) and/or a protease inhibitor (ritonavir/RTV) for 7 days, followed by harvesting to prepare protein lysates. C = control (non-infected PBMCs); 3TC = PBMCs incubated with 3TC; RTV = PBMCs incubated with RTV. **b** Enriched monocytes or CD4^+^ T lymphocytes obtained from whole blood from patients/volunteers were used to produce protein lysates. C = control (blood donors), n = 10; TN = the treatment-naïve patients, n = 7; TNPI = patients under ARV therapy without a protease inhibitor, n = 9; TPI = patients under ARV therapy with a protease inhibitor, n = 9. *p < 0.05; **p < 0.01; ***p < 0.001

PBMCs from healthy volunteers were submitted to HIV infection under different ARV treatment conditions, and the expression of the phosphorylated form of IRE1 (P-IRE1), one of the major sensors of the UPR pathway, was analyzed. As for eIF2α, the phosphorylation of IRE1 also represents its activation. One of the most important downstream effects of IRE1 activation is the splicing of XBP1 mRNA, which, in this condition, will be translated as an important transcription factor that induces the expression of ER stress-response genes. In the present study, in *in vitro* assays, HIV infection was able to induce P-IRE1 expression in PBMCs, regardless of the presence or absence of ARV drugs (Fig. 3a). However, this upregulation of P-IRE1 was significantly higher when the cells were incubated with ARV drugs. Curiously, in this case, treatment restricted to lamivudine was shown to lead to higher expression of P-IRE1 (*p* < 0.01) than treatment with ritonavir did (*p* < 0.05).

As observed in the *in vitro* assays, P-IRE1 expression levels in monocytes and CD4^+^ T lymphocytes from HIV-infected patients were higher compared with levels in cells obtained from healthy individuals (Fig. 3b). Despite the change in the protein expression profile of the infected cells relative to the control group, in which the expression of P-IRE1 was not detected, the expression of this protein was significantly higher only in CD4^+^ T lymphocytes from patients under ARV therapy (*p* < 0.001). A comparison between the *in vitro* and *in vivo* conditions again shows that HIV infection upregulated the expression of P-IRE1 but that there was also a synergistic effect of the ARV drugs. The results presented here corroborate previous reports showing that protease inhibitors trigger the UPR and also present new evidence that other drugs, such as the nucleoside transcriptase inhibitor used in this work, can lead to activation of this pathway.

### ATF6 is cleaved in HIV-infected cells

**Fig. 4:**
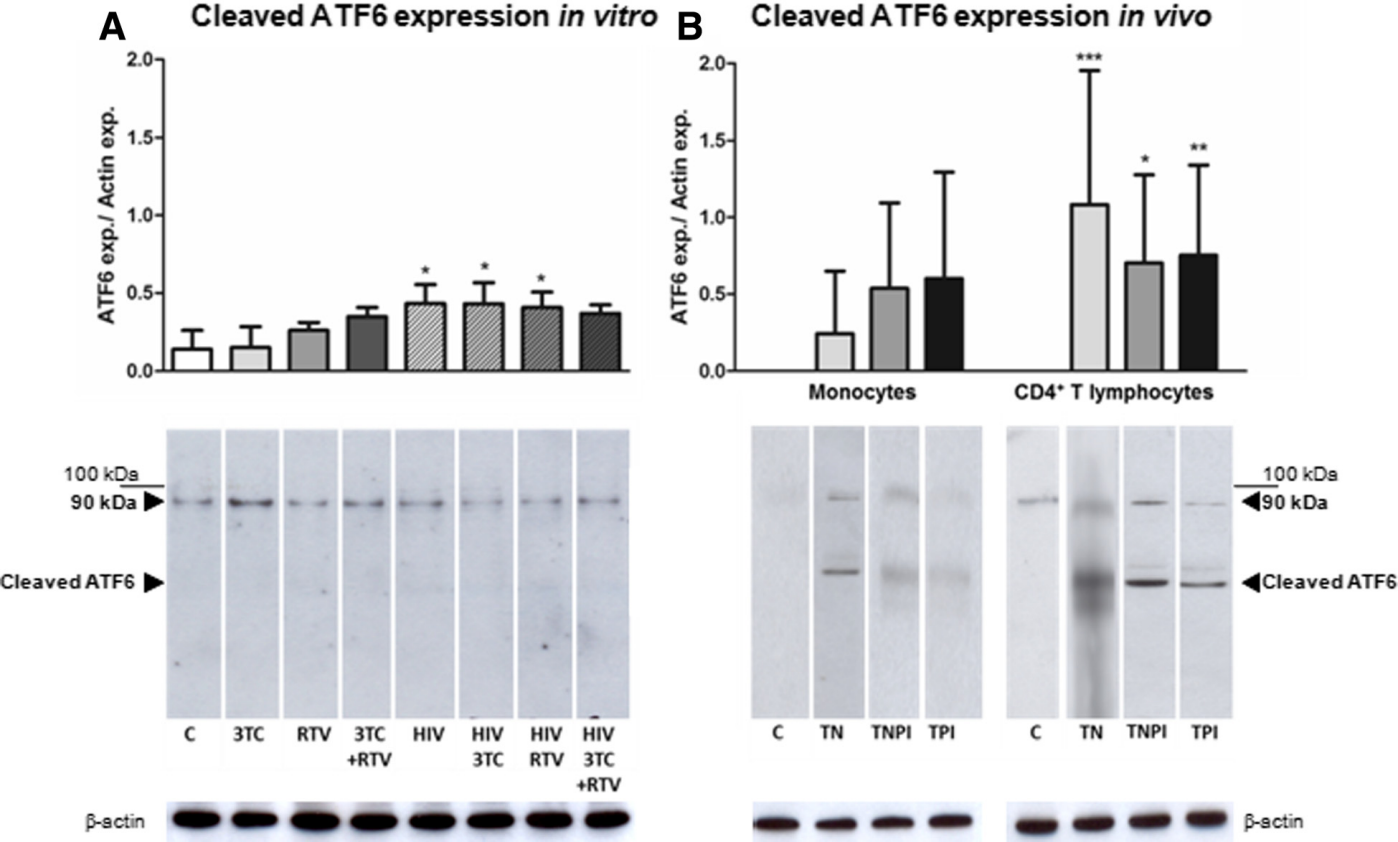
The cleaved form of ATF6 has higher expression in HIV-infected cells. The expression of the uncleaved (90 kDa) and cleaved (50 kDa) ATF6 molecules was determined by Western blotting (bottom panels) using anti-ATF6 antibodies. Actin was used as a loading control. The intensity of the resulting bands was measured using ImageJ, and the cleaved ATF6/actin ratio is shown in the upper panels. Each lane shown in the Western blots is representative of cells under the same infectious and pharmacological conditions *in vitro* or *in vivo*. The bars represent mean ± standard deviation (SD) of of the expression of the cleaved form in each group. **a** PBMCs were infected with the HIV-1 R5 isolate Ba-L, maintained free of ARV drugs or incubated with a nucleoside reverse transcriptase inhibitor (lamivudine/3TC) and/or a protease inhibitor (ritonavir/RTV) for 7 days, followed by harvesting to prepare protein lysates. C = control (non-infected PBMCs); 3TC = PBMCs incubated with 3TC; RTV = PBMCs incubated with RTV. **b** Enriched monocytes or CD4^+^ T lymphocytes obtained from whole blood from patients/volunteers were used to produce protein lysates. C = control (blood donors), n = 10; TN = the treatment-naïve patients, n = 7; TNPI = patients under ARV therapy without a protease inhibitor, n = 9; TPI = patients under ARV therapy with a protease inhibitor, n = 9. *p < 0.05; **p < 0.01; ***p < 0.001

ATF6 is one of the three major sensors of the UPR pathway, and it is activated after cleavage that occurs in the Golgi apparatus. Cleaved ATF6 is a transcription factor that leads to increased expression of UPR-related genes, including the gene encoding the chaperone BiP. In the current study, Western blotting assays were performed with an antibody capable of detecting the cleaved and non-cleaved forms of ATF6. In both *in vitro* and *in vivo* assays, a larger amount of ATF6 was cleaved when cells were infected with HIV, and ARV therapy did not significantly affect the expression profile of this protein. In particular, the expression of cleaved ATF6 in HIV-infected PBMCs was significantly higher (*p* < 0.05) compared with expression in non-infected PBMCs (Fig. 4a).

Monocytes from HIV-infected patients showed higher, but not statistically significantly higher, expression of the cleaved form of ATF6 compared with monocytes from healthy individuals (Fig. 4b). A similar phenotype was observed in CD4^+^ T lymphocytes, although the expression of cleaved ATF6 was significantly higher in cells from HIV-infected patients. Moreover, treatment-naïve patients showed slightly higher expression of this protein (*p* < 0.001) compared with patients under ARV therapy without a protease inhibitor (*p* < 0.05) or with a protease inhibitor (*p* < 0.01). These ATF6 cleavage results from both the *in vitro* and the *in vivo* assays suggest that HIV is responsible for the activation of this UPR pathway sensor.

## Discussion

Aiming to contribute to a better understanding of the UPR activation caused by HIV, this study focused on HIV target cells under variable ARV treatment settings under both *in vitro* and *in vivo* conditions.

A general increase in BiP expression was observed in *in vitro* HIV-infected PBMCs as well as in CD4^+^ T cells and monocytes from HIV patients (Fig. 1). BiP is an ER chaperone involved in protein folding and also in UPR activation [15]. Certain viral infections have already been reported to lead to increased BiP expression, including members of the *Flaviviridae* family, such as dengue virus (DENV) [16, 17], West Nile virus (WNV) [18], and HCV [19], in addition to CMV [20] and enterovirus 71 (EV71) [21]. Higher BiP expression is a strong indication of ER stress and the need for better folding capacity via UPR activation [1]. In the present study, the absence of significant differences in BiP expression between cells free of pharmacological treatment and ARV-treated cells and the lack of a significant impact of the ARV drugs themselves on non-infected cells strongly suggest that HIV was solely responsible for the stress observed in the ER. In the case of infected cells from patients under ARV therapy, despite their undetectable viral loads, the permanence of stress is probably related to the continuous presence of pro-inflammatory cytokines present under these pharmacological conditions [22].

As found for BiP, ATF6 showed higher expression of its activated form in HIV-infected cells, regardless of the pharmacological conditions (Fig. 4). Activated ATF6 leads to higher expression of protein folding-related genes, such as BiP [23]. ATF6 activation has already been described in cells infected with EBV [24], HCV [19], DENV [17], lymphocytic choriomeningitis virus (LCMV) [25], or WNV [18]. Considering the role of ATF6 as a transcriptional activator of BiP expression, it is reasonable to relate the greater abundance of its cleaved state in infected cells to the consequent increase in BiP expression to enhance the protein-folding capability of the ER lumen [26].

The other UPR sensors, P-eIF2α and IRE1, showed different expression profiles than those observed for BiP and ATF6. For these two molecules, the pharmacological conditions seemed to be an important factor in modifying protein expression profiles between different cell groups (Figs. 2 and 3). More specifically, a tendency toward increased expression of P-eIF2α was observed in non-infected PBMCs treated with the protease inhibitor RTV, regardless of the presence of a second drug, compared with cells not submitted to ARV drugs. In addition, when non-treated infected PBMCs were compared with non-treated cells from the non-infected group, increased levels of P-eIF2α were observed. However, for both comparisons, the differences were not significant. Among HIV-infected PBMCs, significantly higher expression of P-eIF2α was found in cells submitted to ARV-mediated pharmacological control. Under *in vivo* conditions, CD4^+^ T lymphocytes had significantly higher expression of P-eIF2α than did monocytes, particularly among the cells obtained from patients undergoing ARV treatment. This scenario is interesting because when PERK phosphorylates eIF2α, the latter factor is deactivated. Simultaneously, eIF2α phosphorylation also increases translation of ATF4, which induces expression of several UPR-related genes, including BiP. These event leads to an attenuation of protein translation, decreasing the load of new proteins to be folded in the ER lumen [27].

Accumulation of the phosphorylated form of eIF2α in infected cells must be considered as a strategy to avoid an excess of proteins to be folded in the ER lumen. Caselli et al. have previously suggested that P-eIF2α can promote a favorable environment for HIV replication [10]. The presence of P-eIF2α may also lead to virus reactivation from reservoirs, such as gut lymphoid tissues. Considering the role of P-eIF2α in disrupting HIV latency, the results obtained from patients undergoing ARV therapy suggest a propitious environment for HIV according to its described role in previous studies, which is an unexpected perspective on cells under pharmacological control. Nevertheless, it is already known that ARV drugs are capable of inducing ER stress and UPR activation, which raises the possibility that this higher UPR activation signal in treated cells compared with cells without treatment is due to pharmacological UPR activation.

The different expression profiles between monocytes and CD4^+^ T lymphocytes could be related to discrepancies in the HIV replication cycle between the two cell types. It is well known that monocytes and macrophages are very resistant to HIV infection and its cytopathic effects in comparison with CD4^+^ T lymphocytes [28]. This refractory behavior favors latency and the role of monocytes and macrophages as reservoirs [29]. Even in patients with no detectable viral load, it is possible to recover and reactivate HIV replication in these cell types [30]. Considering that HIV plays a role in eIF2α phosphorylation, the lack of significant eIF2α expression in monocytes may be related to the less efficient HIV replication in these cells. In contrast, activated CD4^+^ T lymphocytes are the most permissive cellular target of HIV, and when infected, these cells can die through caspase-3-dependent apoptosis [31]. However, the frequency of infected lymphocytes under clinical conditions is relatively low, as permissive CD4^+^ T lymphocytes represent less than 5 % of the whole lymphocyte population [32]. These data suggest that the progressive loss of CD4^+^ T lymphocytes can occur through other mechanisms. In fact, it has already been described that approximately 95 % of the cell death in HIV patients occurs through a caspase-1-dependent and highly inflammatory mechanism called pyroptosis, which happens in less permissive cells [33]. A highly inflammatory environment is established even in patients submitted to ARV therapy and with undetectable viral loads [22]. From the beginning of an HIV infection, there is a massive depletion of memory CD4^+^ T lymphocytes in the gastrointestinal mucosa [34]. This depletion facilitates the translocation of microbial products from the gut to the blood and creates a constant source of immune activation and inflammation that persists after ARV treatment [35]. Therefore, CD4^+^ T lymphocytes in HIV patients are under constant metabolic stress, and the presence of P-eIF2α is an indication of this condition.

As mentioned before, in the current study, P-IRE1 expression showed a similar profile to P-eIF2α expression (Fig. 3). IRE1 in its phosphorylated form indicates activation of this UPR sensor [36]. As IRE1 is responsible for the selective splicing of XBP1 mRNA (generating sXBP1), it is expected that IRE1 activation leads to a higher amount of sXBP1 and further expression of protein degradation-related genes [37], decreasing the protein-folding workload in the ER. IRE1 phosphorylation has already been described in avian coronavirus infectious bronchitis virus (IBV) [38], EBV [24], CMV [39], HCV [40], DENV-1 [17], and WNV [18] infections. The state of protein degradation created by P-IRE1 in the cell is not advantageous for viral replication. To evade this cellular stress response, certain viruses, such as CMV and HCV, can suppress the activity of sXBP1 and avoid the induction of its target genes [39, 40]. In IBV-infected cells, IRE1 activation serves as a survival factor during coronavirus infection, protecting cells from apoptosis [38]. The higher expression of P-IRE1 in treated patients with undetectable viral loads and the lower expression in treatment-naïve individuals observed in the current study are in agreement with activation of the IRE1 sensor serving as a cellular defense mechanism. However, further studies are necessary to elucidate whether HIV also employs strategies to prevent possible losses of its replication under these conditions.

**Fig. 5:**
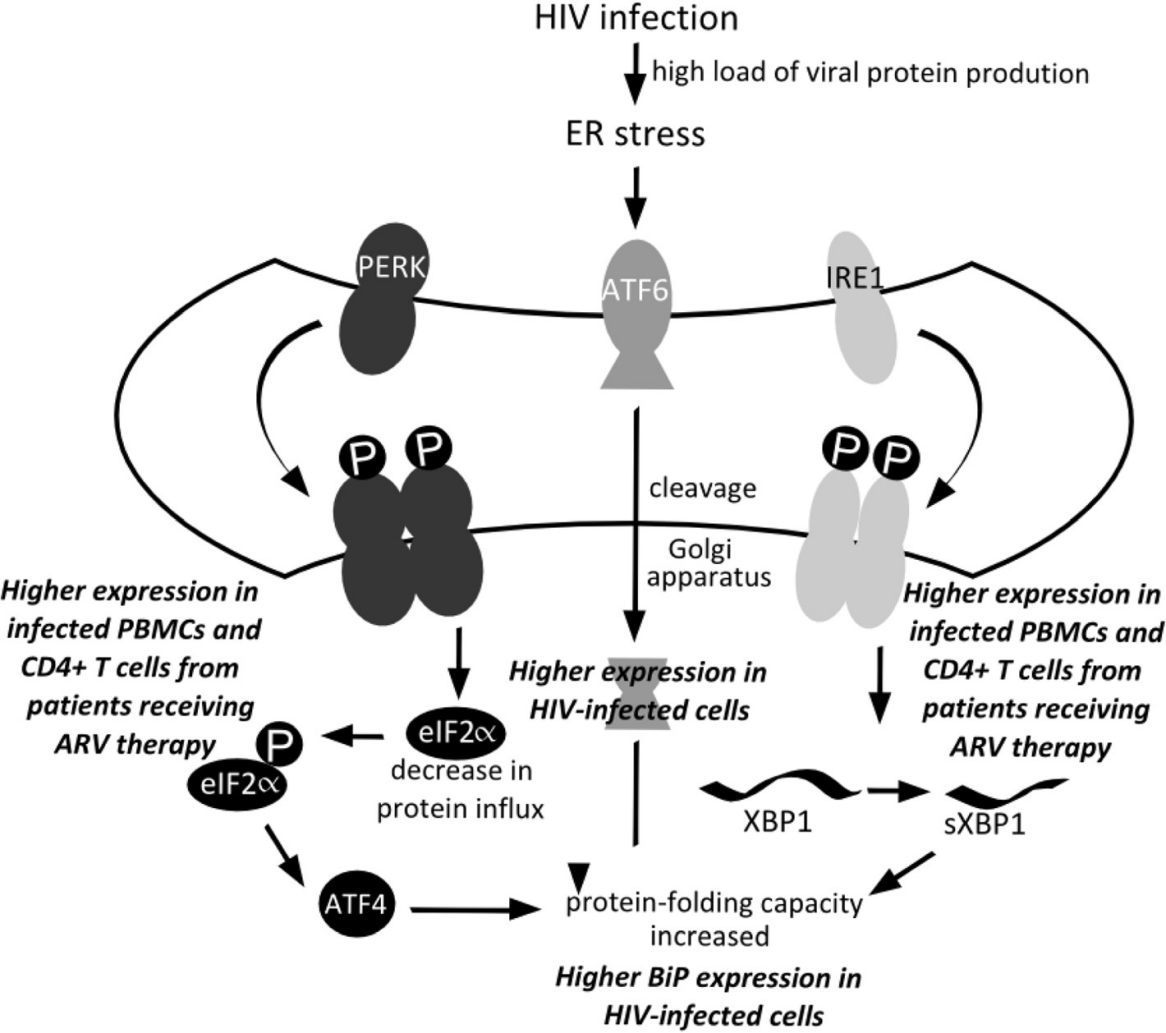
The UPR is activated in HIV-infected cells. The expression of BiP and cleaved ATF6 is increased in HIV-infected cells, regardless of ARV therapy. Additionally, the PERK-related factor eIF2α and the sensor IRE1 are phosphorylated in HIV-infected cells under ARV treatment conditions. Phosphorylation and activation of these molecules are known to lead, respectively, to selective translation of ATF4 and XBP1 splicing, both contributing to a higher protein-folding capacity in the ER

The entire scenario of UPR activation presented in this study revealed higher expression of ATF6 and BiP in PBMCs as well in cells from patients, regardless of ARV treatment, whereas activation of the other two UPR sensors, PERK and IRE1, was ARV treatment dependent (Fig. 5). For infected cells not submitted to ARV therapy, ATF6 and BiP presented higher expression levels. For infected cells under ARV treatment, including PBMCs cultured *in vitro* and monocytes/CD4^+^ T lymphocytes from volunteer patients, the highest levels of UPR activation were present, triggering the activation of the three pathway sensors PERK, represented by P-eIF2α, ATF6 and IRE1. These results suggest that in an environment with active HIV replication, the UPR pathway is modulated by the virus, although the exact mechanisms are not completely understood.

## Conclusions

The results presented here indicate that HIV affects UPR activation through its three major sensors: PERK, IRE1, and ATF6. HIV-infected cells upregulate these UPR-related proteins in a way that seems to be independent of the ARV treatment to which they are submitted. However, a synergistic pharmacological effect appears to occur, as the drugs do not show the same influence on the protein expression profile of non-infected cells under both *in vitro* and *in vivo* conditions. These findings suggest two possible concurrent mechanisms: the interference of HIV itself and the pharmacological effects of the ARV drugs, both acting as activators of the UPR. This report presents new evidence of how HIV modulates the UPR pathway to enhance its own replication and secure infection success, and also raises the promising possibility of using this pathway as a drug target in the development of new antiviral therapies.

## Methods

### Ethics statement

This study was approved by the Internal Review Board of Centro de Hematologia e Hemoterapia de Santa Catarina (HEMOSC, Florianópolis, Brazil), Hospital Regional Dr. Homero de Miranda Gomes (HRHMG, São José, Brazil), and Centro de Testagem e Aconselhamento (CTA, São José, Brazil) and by the Ethical Committees of Universidade Federal de Santa Catarina (UFSC, Florianópolis, Brazil) and Fundação Oswaldo Cruz (FIOCRUZ, Rio de Janeiro, Brazil). All study subjects provided written informed consent prior to enrollment.

### Study population

The study sample consisted of 35 individuals. Eligible volunteers included individuals over18 years old who were recruited at HEMOSC, HRHMG, and CTA. The individuals were selected from April 2011 to July 2012. The volunteers were then arranged into four distinct groups: i) HIV-negative blood donors (C) (n = 10), ii) treatment-naïve HIV-positive patients (TN) (n = 7; mean viral load: 3.5 ± 0.7 log_10_ copies/ml), iii) HIV-positive patients under ARV therapy without a protease inhibitor (TNPI) (n = 9; undetectable viral load), and iv) HIV-positive patients under ARV therapy including a protease inhibitor (TPI) (n = 9; undetectable viral load). Infections by Epstein-Barr virus (EBV), cytomegalovirus (CMV), hepatitis C virus (HCV), herpes simplex 1 virus (HSV-1), or human herpes virus 8 (HHV-8), or any other acute infections were considered as exclusion criteria for any of the groups described. The clinical data from the HIV-positive patients were accessed through their medical records.

### Sample collection and processing

For the *in vivo* assays, whole-blood samples from the enrolled volunteers were collected in EDTA tubes. CD4^+^ T lymphocytes and monocytes were then isolated from peripheral blood mononuclear cells (PBMCs) using the RosetteSep Human CD4^+^ T Cell Enrichment Cocktail and RosetteSep Human Monocyte Enrichment Cocktail kits (STEMCELL Technologies), respectively. The cell purity was determined using fluorochrome-labeled anti-CD3, anti-CD4, and anti-CD14 antibodies (BD Pharmingen), a FACSCalibur flow cytometer (Becton Dickinson), and the software FlowJo 8.6.3 (© Tree Star) (Additional file 2: Figure S1).

### HIV infection assay

PBMCs from 3 healthy donors were obtained by density gradient centrifugation (Hystopaque, Sigma) of buffy-coat preparations. These PBMCs were resuspended in RPMI 1640 (LGC Bio) supplemented with 10 % heat-inactivated fetal bovine serum (FBS; HyClone, Logan, UT), penicillin (100 U/mL), streptomycin (100 μg/mL), 2 mM glutamine and 10 mM HEPES, followed by stimulation with 5 μg/mL phytohemagglutinin (PHA; Sigma) for 2–3 days and further maintenance in culture medium containing 5 U/mL recombinant human interleukin-2 (Sigma). The PBMCs were then infected with the HIV-1 R5 isolate Ba-L using 5–10 ng/mL p24 antigen. After 2 h of incubation, the cells were washed to remove excess virus. Culture medium was added to the infected PBMCs, followed by treatment with ARV drug(s), which lasted throughout the infection period. Next, HIV-1 replication was evaluated in the cell culture supernatants 7 days after infection using a commercial ELISA kit (ZeptoMetrix Co.). The cells were divided into 4 groups: i) untreated cells, ii) cells treated with a nucleoside reverse transcriptase inhibitor (lamivudine/3TC, 1 μM), iii) cells treated with a protease inhibitor (ritonavir/RTV, 10 μM), and iv) cells treated with 3TC + RTV. Additionally, non-infected cells submitted to the 4 different conditions described were used as controls.

### Preparation of cellular protein lysates

Cellular protein lysates were prepared from 1 × 10^6^ CD4^+^ T lymphocytes or monocytes from enriched subpopulations using the Ambion® PARIS™ system (Life Technologies). A protease inhibitor cocktail (Sigma-Aldrich) was added to the resulting lysates, which were then stored at −20 °C. For the *in vitro* assays, the PBMCs were suspended in a lysis buffer composed of 20 mM Tris–HCl, pH 7.5, and a protease and phosphatase inhibitor cocktail (Sigma-Aldrich). After incubation for 10 min at 4 °C, followed by centrifugation (20,000 × g, 10 min, 4 °C), the supernatants were collected and stored at −70 °C. Protein quantification was then performed via the bicinchoninic acid (BCA) method using a Micro BCA Protein Assay Kit (Pierce), with the actual measurement performed by spectrophotometry at 562 nm using an Infinite M200 microplate reader (Tecan).

### Western blotting

In total, 10 μg of each cellular protein lysate sample was separated by 12 % SDS-PAGE and electro-blotted onto a nitrocellulose membrane (GE Healthcare) soaked with blotting buffer (25 mM Tris, 192 mM glycine, and 20 % methanol, pH 8.3). Subsequently, the membranes were incubated for 1 h at 25 °C with rabbit anti-actin (ab8227, Abcam), mouse anti-BiP (ab96483, Abcam), rabbit anti-P-eIF2α (ab4837, Abcam), rabbit anti-P-IRE1 (ab48187, Abcam), or mouse anti-ATF6 (ab11909, Abcam). The blots were then incubated with an HRP-conjugated anti-mouse or anti-rabbit total immunoglobulin (respectively, ab6728 or ab6721, Abcam) for 1 h at 25 °C. The detection was performed using the ECL reagent (Pierce) and exposure to radiographic films (GE Healthcare), the development of which was performed using an automated SRX-101A processor (Konica Minolta).

### Protein expression evaluation by densitometry

Quantitative analysis of the data obtained from the Western blotting experiments was performed by band densitometry using the software ImageJ (http://rsbweb.nih.gov/ij/).

### Statistical analysis

The data were presented as mean ± standard deviation (SD). The results were plotted and submitted to statistical analysis through the ANOVA parametric test. Observations were considered statistically significant when *p* < 0.05. The statistical analyses were performed using the software GraphPad InStat®, and the graphs were drawn with GraphPad Prism, version 5.

## Additional files

Additional file 1: Table S1. HIV-1 replication in PBMCs from healthy donors, in the presence or not of antiretrovirals. Additional file 2: Figure S1. Purity of CD4^+^ T lymphocytes and monocytes measured by flow cytometry.
