# Supplementary material for: HIV infection and antiretroviral therapy lead to unfolded protein response activation
Source: Virol J. 2015 May 15;12:77. doi: 10.1186/s12985-015-0298-0 (PMC4455982; doi:10.1186/s12985-015-0298-0)
Supplement: Additional file 1: Table S1. — HIV-1 replication in PBMCs from healthy donors, in the presence or not of antiretrovirals. [file 12985_2015_298_MOESM1_ESM.docx]

Table 1. HIV-1 replication in PBMCs from healthy donors, in the presence or not of antiretrovirals.

|  | HIV-1 p24 Ag release, ng/ml (% inhibition of viral replication)^*^ | | | | |
| --- | --- | --- | --- | --- | --- |
|  |  |  | ARV^**^ | | |
| Donor | Nil |  | 3TC^**^ | RTV^**^ | 3TC+RTV |
| 1 | 23.71 |  | 0.95 (95.9) | 0.07 (99.7) | 0 (100) |
| 2 | 147.87 |  | 2.07 (98.6) | 0.92 (99.4) | 0.02 (99.9) |
| 3 | 181.32 |  | 0 (100) | 0 (100) | 0 (100) |
| *Measured in the cell culture supernatants by ELISA, 7 days after infection.  ** ARV, antiretroviral; 3TC, lamivudine, 1 μM; RTV, ritonavir, 10 μM. | | | | | |
