# Supplementary figures and images for: HIV infection and antiretroviral therapy lead to unfolded protein response activation
Source: Virol J. 2015 May 15;12:77. doi: 10.1186/s12985-015-0298-0 (PMC4455982; doi:10.1186/s12985-015-0298-0)

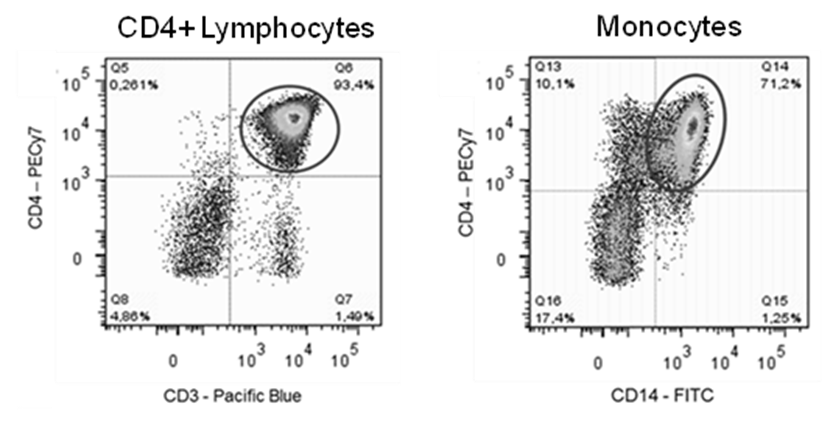

Supplement: Additional file 2: Figure S1. — Purity of CD4+ T lymphocytes and monocytes measured by flow cytometry. [file 12985_2015_298_MOESM2_ESM.tiff]
